# Effects and Mechanisms of Imperatorin on Vitrified Mouse Oocytes

**DOI:** 10.3390/ani15050661

**Published:** 2025-02-25

**Authors:** Yuan Feng, Mengyuan Zhang, Wenqing Yuan, Dan Zhao, Zhixuan Luo, Zihui Tang, Yongheng Wang, Ming Cang

**Affiliations:** The State Key Laboratory of Reproductive Regulation and Breeding of Grassland Livestock, Inner Mongolia University, Hohhot 010030, China; 13368970579@163.com (Y.F.); 13674754350@163.com (M.Z.);

**Keywords:** imperatorin, vitrification, mouse oocytes, oxidative stress, antioxidant, mitochondria, apoptosis, glutathione, reactive oxygen species, cryopreservation

## Abstract

Imperatorin (IMP) is a naturally occurring furanocoumarin with beneficial biological activities such as anticancer, antioxidant, and neuromodulatory properties. Currently, the protective effects and mechanisms of IMP on oxidative stress experienced by mouse oocytes after vitrification–thawing remain unclear. To investigate the influence of IMP on mouse oocyte development after vitrification–thawing, we added different concentrations of IMP to the vitrification and thawing media. Results indicated that the addition of 40 μM IMP enhanced post-thaw fertilization capacity, reduced intracellular reactive oxygen species (ROS) levels, and increased intracellular glutathione (GSH) levels. IMP also improved mitochondrial health by alleviating the decrease in mitochondrial membrane potential (MMP) and enhancing mitochondrial distribution. IMP reduced intracellular ROS levels by affecting the transcription of the antioxidant genes *SOD2*, *NRF2*, and *HO-1* and enhancing SOD activity. It also elevated GSH levels via *GPX1*, improved mitochondrial function, and decreased early apoptosis through *Bcl-2.* In conclusion, IMP enhanced ovum health through the alleviation of oxidative stress. The present study provides useful information for further exploration of the molecular mechanisms of IMP in female reproductive cells and offers a novel approach for the improvement of vitrification technology.

## Simple Summary

When precious species are about to die out or excellent strains need to be preserved, we want to use technology to preserve their gametes so that they can be used directly when needed in the future. The damage caused by vitrification of frozen oocytes needs to be addressed. In this study, the damage to oocytes caused by freezing was minimized by natural chemicals. The results showed that imperatorin reduced the level of oxidative stress and increased mitochondrial activity in mouse oocytes after vitrification freezing. In conclusion, the addition of imperatorin to vitrification freezing solution improved the activity of frozen mouse oocytes and provided new perspectives for the improvement of vitrification freezing technology.

## 1. Introduction

Oocyte cryopreservation by vitrification technology can preserve good strains, as well as protect the genetic material of endangered and extinct species, oocyte cryopreservation technology is of great significance to the preservation of animal germplasm resources, and it has become an important part of the good trait improvement and assisted reproduction technology. Several studies have indicated that vitrification technology is one of the most effective methods for ovum cryopreservation due to its ability to achieve rapid cooling while causing little change in osmotic pressure and eliminating the formation of ice crystals [1]. Vitrification technology has been widely applied in the preservation of animal gametes and embryos [2,3,4]. However, the development rate of post-vitrified oocytes remains a key issue [5]. The toxic effects of cryoprotective agents (CPAs) [6], oxidative stress induced by excessive accumulation of intracellular reactive oxygen species (ROS) [7], and osmotic stress triggered by low temperature [4] will result in the lowering of the development potential of oocytes. In particular, oxidative stress induced by excessive ROS accumulation has garnered widespread attention.

During the ovum vitrification process, osmotic CPAs such as dimethyl sulfoxide (DMSO) cause the endoplasmic reticulum to release large amounts of Ca^2+^, which are absorbed by mitochondria. This leads to prolonged opening of the mitochondrial permeability transition pore (mPTP), thereby causing the loss of mitochondrial membrane potential (MMP) and adenosine triphosphate (ATP) [8] and a further rise in ROS levels. Low-temperature stimulation also aggravates the production of ROS [9]. During the vitrification process, which is performed in vitro, blue light used in the laboratory environment (400–500 nm) can also cause oxidative injury to cells by altering enzymes in the respiratory chain [10,11]. It has been reported that microscopy accounts for 95% of the damaging radiation to which the cells are exposed [12]. High pH levels and oxygen concentrations also lead to an increase in oxidase activities, thereby inducing an increase in intracellular O_2_ levels [13].

Under physiological conditions, ROS can control key biological processes by serving as signaling molecules. However, when ROS production exceeds the maximum ROS elimination capacity of the antioxidant defense system, intracellular redox balance is disrupted, causing elevated oxidative stress levels. This leads to increased oxidative DNA damage [14], DNA strand breakage [15], and oxidative protein damage [16], thus weakening the developmental capacity of oocytes. Therefore, recent studies have focused on the use of small-molecule antioxidants for the protection of oocytes during the cryopreservation process [17,18,19].

Imperatorin (IMP) is a naturally occurring furanocoumarin that mainly exists in Danggui (Radix Angelicae Sinensis) [20], Hongjingtian (*Rhodiolae crenulatae* Radix et Rhizoma) [21], and fennel (*Foeniculum vulgare* Mill.) [22]. In particular, Danggui is a commonly used Chinese medicinal herb, and IMP obtained from its extract possesses antioxidant, immunomodulatory anticancer, and neuromodulatory properties that can be used for the treatment of gynecological diseases, cardiovascular diseases, and neurological disorders [23,24,25]. IMP has proven effective in regulating the expression of superoxide dismutase (SOD), xanthine oxidase (XOD), and nicotinamide adenine dinucleotide phosphate (NADPH) [26], reducing the generation of ROS [27], and protecting mitochondrial function [28].

## 2. Materials and Methods

### 2.1. Experimental Animals

This study used female Kunming (KM) mice aged six weeks and male KM mice aged eight weeks. The mice were purchased from SPF Biotechnology Co., Ltd. (Shanghai, China). All animals were reared at the Animal Center of the State Key Laboratory of Reproductive Regulation & Breeding of Grassland Livestock at Inner Mongolia University in accordance with the relevant Chinese national standard for the care and use of laboratory animals. The rearing environment was controlled at 21 ± 2 °C, a relative humidity of 40–70%, and a 12 h light (8:00–20:00)/12 h dark (20:00–8:00) cycle.

### 2.2. Collection of Oocytes

Female mice were injected intraperitoneally with 10 IU of pregnant mare serum gonadotropin (PMSG; Ningbo Second Hormone Factory, Ningbo, China), reared in a sterile filter-top cage at 25 °C for 48 h, and injected intraperitoneally with 10 IU of human chorionic gonadotropin (hCG; Ningbo Second Hormone Factory, China) to induce superovulation. The mice were euthanized by cervical dislocation at 13–16 h after hCG injection. Ovaries and fallopian tubes were obtained by dissection of the mice and placed in M2 medium (Sigma-Aldrich, St. Louis, MO, USA). After the ampullary part of the fallopian tubes had been cut open with a scalpel, the cumulus mass was removed by traction, immediately exposed to HEPES-buffered medium containing 80 IU/mL hyaluronidase (Vitrolife, Beijing, China), and mixed thoroughly with a pipette for the removal of cumulus and corona radiata cells from the cumulus–oocytes complex (COCs). The degree of oocyte maturation was assessed by observation of the first polar body (PB1) under a stereo microscope. Oocytes at the metaphase II (MII) stage with good morphology (normal oocytes are usually round or oval in shape, moderately sized, and the zona pellucida of good quality oocytes should be intact, uniform, and undamaged; the cytoplasm should be clear and free of granular impurities; the presence of polar bodies should be clearly recognizable) and a transparent and uniform cytoplasm were selected [29,30], rinsed in M16 medium (Sigma-Aldrich, Saint Louis, MO, USA) that had been pre-equilibrated in a CO_2_ incubator for 12 h, and used for further experimentation.

Oocytes at the germinal vesicle (GV) stage were collected using a procedure similar to that described above. At 48 h after injection of PMSG, GV oocytes were obtained from the ovarian follicles by dissection.

### 2.3. Vitrification and Thawing of MII Oocytes

MII oocytes were divided into negative control, positive control, and experimental groups. Negative controls were oocytes freshly removed from mice without any treatment. Oocytes of the positive control group were placed in Kitazato equilibration medium (Kitazato, Fuji, Japan) containing 7.5% ethylene glycol, 7.5% DMSO, and 20% human serum albumin for 5 min. When the size of the perivitelline space of the oocytes was similar to that of the zona pellucida (ZP), i.e., oocyte equilibration had been completed, the oocytes were transferred using a mouth-controlled Pasteur pipette to Kitazato vitrification medium (Kitazato, Japan) containing 15% ethylene glycol and 15% DMSO at room temperature. After treatment for 60 s, samples were loaded onto Cryotop devices (RUBAL Shandong, China) using a mouth-controlled pipette, with each Cryotop device loaded with 1 μL of liquid containing up to 10 oocytes.

Cryotop devices stored in liquid nitrogen for one week were placed in Kitazato thawing medium (Kitazato, Fuji, Japan) for treatment at 25 °C for 60 s, placed in dilution solution for 3 min, then washed twice in washing solution for 5 min each time. When the thawing process was complete, the oocytes were transferred to M16 medium and incubated in a high-humidity incubator containing 5% CO_2_ and 95% air. After incubation for 1 h, the oocytes of each group were collected for in vitro fertilization (IVF) or staining.

For the experimental group, the vitrification procedure was identical to that of the positive control group. In the pre-experiment, we used 1 μM, 5 μM, 50 μM, 100 μM, and 1000 μM and found that the effect is better at 50 μM. Therefore, the equilibration medium, vitrification medium, and solutions for thawing, dilution, rinsing, and washing were, respectively, supplemented with 20 μM, 40 μM, and 60 μM IMP (MedChemExpress, Shanghai, China).

### 2.4. Sperm Collection and IVF

Male KM mice aged eight weeks were euthanized by cervical dislocation. The entire epididymis was removed, folded on clean filter paper to leave only the epididymal tail exposed, and cut open horizontally with iris scissors. Semen was squeezed out, collected in a glass pipette by dipping, and rapidly transferred to T6 medium that had been pre-equilibrated overnight and contained 10 mg/mL bovine serum albumin (BSA; Coolaber, Beijing, China). Sperm screening selects for use by briefly centrifuging the sperm and selecting those that swim to the supernatant. Sperm concentration was diluted to 1 × 10^6^/mL, and the sperm culture was incubated in a CO_2_ incubator for 60 min for capacitation.

MII oocytes from each group were separately placed in different HTF media (Sigma-Aldrich, Saint Louis, MO, USA) that had been pre-equilibrated overnight and covered with mineral oil. Spermatozoa were aspirated with 10 μL of screened sperm and added to HTF medium to co-culture with oocytes for 3 h. After incubation, the oocytes were transferred to KSOM medium (Sigma-Aldrich, Saint Louis, MO, USA), where Ksom cultures were placed in 35 mm dishes at 40 μL per liquid and covered with mineral oil, washed for the removal of excess sperm, and cultured for 120 h. Early embryonic development was observed at 24 h, 96 h, and 120 h.

### 2.5. In Vitro Maturation (IVM)

GV oocytes of the control group were cultured in 20 μL of M16 culture covered with mineral oil (Aibei, Nanjing, China) and cultured in an incubator containing 5% CO_2_ at 37 °C. After 16–20 h [31], the oocytes were examined under an inverted microscope. Oocytes with PB1 were selected for further study [32].

A similar procedure was adopted for the experimental group, except that the M16 medium was separately supplemented with 20 μM, 40 μM, and 60 μM IMP.

### 2.6. Measurement of ROS and GSH

The H_2_O_2_ group was incubated in 1000 μM H_2_O_2_ for 1 h, as with each of the remaining groups. Ten oocytes of each group were separately cultured in CZB medium containing 10 μM 2′,7′-dichlorodihydrofluorescein diacetate (DCFH-DA; MedChemExpress, Shanghai, China) for 30 min, rinsed thrice with 0.1% PVA-PBS (*w*/*v*), and photographed under a fluorescence microscope (Ex/Em = 488/525 nm). Photographs were saved in .tif format.

Ten oocytes of each group were separately incubated in CZB medium containing 10 μM monochlorobimane (MedChemExpress) for 30 min and subsequently rinsed thrice with 0.1% PVA-PBS (*w*/*v*). The oocytes were photographed under a fluorescence microscope (λ_ex_ = 380 nm, λ_em_ = 470 nm), with photographs saved in .tif format.

### 2.7. Measurement of Mitochondrial Distribution

Ten oocytes of each group were placed in 0.1% PVA-PBS (*w*/*v*) containing 0.2 μM MitoTracker^TM^ Deep Red dye and incubated in a CO_2_ incubator at 37 °C for 30 min. Subsequently, the oocytes were washed thrice with PVA-PBS and incubated with PVA-PBS drops containing 2.5 µg/mL Hoechst 33342 (Sigma-Aldrich, USA) in the dark at room temperature for 10 min. Stained oocytes were transferred with a mouth-controlled Pasteur pipette to a glass bottom culture dish for confocal laser microscopy (NEST, Palo Alto, CA, USA) and photographed under a confocal laser microscope (Nikon, Tokyo, Japan). Oocytes that were uniformly red were considered normal and those with distinct mitochondrial aggregates in the center or along the borders were considered abnormal. The ratio of negative oocytes to the final white fraction of the total number of oocytes was calculated for comparison.

### 2.8. Zona Pellucida Hydrolysis Time Measurement

ZP hardness was measured using a previously described method, with certain modifications [33]. Oocytes were transferred to PBS-PVA, washed by pipetting, and transferred to PBS-PVA in which 50 μL of 0.5% (*w*/*v*) pronase solution (Aladdin, Shanghai, China) had been added. Dissolution of ZP was continuously observed under an inverted microscope. The oocytes were initially observed once every 30 s. When obvious thinning of ZP had occurred, observations were made once every 5 s until complete dissolution of ZP. The ZP dissolution time for each ovum was recorded. Hydrolysis times of oocyte zona pellucida in the fresh group were normalized, and multiplicative differences in hydrolysis times in the other groups were compared separately.

### 2.9. Annexin-V Staining

Early apoptosis was detected with the Annexin-V staining kit (Beyotime Biotechnology, Haimen, China). Oocytes were added to 195 μL of binding buffer containing 5 μL of Annexin-V-FITC and incubated in the dark at room temperature for 20 min. After washing thrice in PBS-PVA, the oocytes were placed in a confocal laser microscopy culture dish and immediately examined under a confocal laser microscope.

### 2.10. Measurement of MMP

Oocytes were treated with 2 μM 5,5′,6,6′-tetrachloro-1,1′,3,3′-tetraethyl-imidacarbocyanine iodide (Beyotime, Shanghai, China) at 37 °C for 2 h and subsequently washed thrice in PBS-PVA. Images were captured under a fluorescence microscope (Nikon, Tokyo, Japan) for analysis of fluorescence intensity in ImageJ software (v1.54). MMP of the oocytes was calculated as the ratio of red fluorescence intensity to green fluorescence intensity.

### 2.11. Measurement of SOD Activity

SOD activity was measured using an SOD assay kit (Beyotime Biotechnology, Shanghai, China) in accordance with the manufacturer’s instructions. Detection was performed by the WST-8 method. In short, 150 oocytes were mixed with 20 μL of lysis buffer and incubated with reaction buffer for 30 min. Absorbance at 450 nm was measured with a microplate reader, and SOD activity was calculated from the absorbance value.

### 2.12. Cortical Granule Staining

The oocytes from different treatments were collected separately, and the oocytes were fixed in 4% paraformaldehyde at room temperature and protected from light for 50 min, after which they were washed three times using 0.1%PVA-PBS (*w*/*v*) for 10 min each time; the washed oocytes were placed in a permeabilized solution of 0.5% Triton X-100 that was configured in advance and processed at room temperature and protected from light for 2 h; the same 0.1%PVA-PBS (*w*/*v*) was used to wash the oocytes for three times. After washing, the oocytes of each group were placed in 1% BSA and closed overnight at 4 °C; the closed oocytes were placed in working concentration of peanut agglutinin (Sigma-Aldrich, Saint Louis, MO, USA) and incubated for 2 h at room temperature and protected from light; the oocytes were washed with PVA-PBS 3 times, each time for 10 min. After washing with 0.1%PVA-PBS, the slices were sealed, placed in a wet box, and photographed and recorded under a laser confocal microscope.

### 2.13. Real-Time Fluorescence-Based Quantitative PCR

Real-time fluorescence-based quantitative PCR (qRT-PCR) was performed using a previously described method. A total of 120 oocytes of each sample were lysed with the RNAprep Pure Micro Kit (TIANGEN Biotech, Beijing, China), and cDNA was directly synthesized using the PrimeScript^tm^ RT reagent Kit (TaKaRa Bio, Beijing, China). Real-time quantitative PCR was performed using TB Green^®^ Premix Ex Taq^TM^ (TaKaRa Bio, China) on the CFX96 Touch Deep Well Real-Time PCR System (Bio-Rad, Hercules, CA, USA). The 2^−∆∆CT^ method was used to calculate cytoplasmic genes, SOD2, GPX1, HO-1, and NRF2, and mitochondrial genes, Nd2, COX1, COX2, and ATP8 (Table 1). GAPDH was selected as an internal reference gene [34]. Each reaction was performed in three independent replicates.

PCR cycles were run as follows: denaturation at 95 °C for 10 min, followed by 40 cycles of 15 s each at 95 °C and 1 min at 60 °C. Fluorescence was measured after each annealing and extension phase. A melting curve was generated to confirm individual gene-specific peaks and to detect primer/dimer formation by heating samples from 70 °C to 95 °C in 0.5 °C increments with a 10 s residence time at each temperature while fluorescence was continuously monitored.

### 2.14. Statistical Analysis

All statistical analyses were performed using SPSS 22.0 (IBM Corporation, Armonk, NY, USA). The two sets of data were compared using the Shapiro–Wilk test by plotting small sample QQ plots. We used the *t*-test for correlation analysis only if it was consistent with normal distribution and homogeneity of variance, and the ANOVA test should be used only if a normal distribution was observed; otherwise, nonparametric tests such as Mann–Whitney should be used. All data are expressed as mean ± standard deviation (SD). The total number of oocytes or embryos (*n*) used in each group has been illustrated in the figures. Black dots represent measurements for each group. Differences are statistically significant when *p* < 0.05 and *p* < 0.01. Different letters represent significant differences at *p* < 0.05.

## 3. Results

### 3.1. Effects of IMP Supplementation on Mouse Oocytes During IVM

We investigated the effects of IMP supplementation on mouse oocytes during IVM by culturing COCs with different concentrations of IMP (20 μM, 40 μM, and 60 μM). The polar body extrusion (PBE) rate, ROS level, and GSH level of the mouse oocytes were measured at the end of IVM.

The addition of 40 μM IMP to IVM medium significantly increased the maturation efficiency of the mouse oocytes (Table 2; 69.79% (±1.75%) vs. 55.13% (±5.13%)), with the value for 40 μM IMP increased by 18.7% (10.91%) and 19.06% (±0.04%) compared with the values for the addition of 20 μM and 60 μM, respectively.

**Figure 1:**
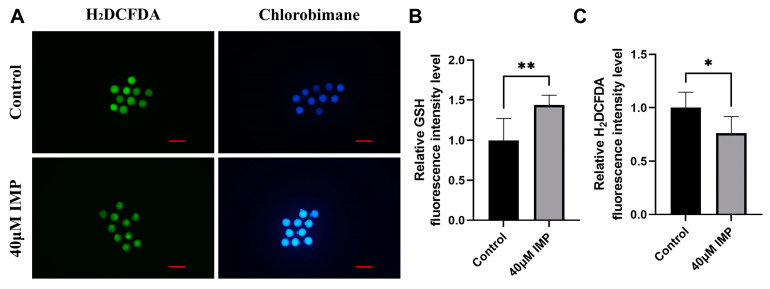
State of mouse oocytes after imperatorin (IMP). (**A**) Representative stains of reactive oxygen species (ROS) and glutathione (GSH) after in vitro maturation (IVM); (**B**,**C**) quantitative analysis of measured ROS and GSH levels in mature mouse oocytes of control group (*n* = 70) and 40 μM IMP group (*n* = 70), R = 7; *: *p* < 0.05; **: *p* < 0.01.

The addition of IMP to IVM medium improved the ROS and GSH levels of mouse oocytes. Figure 1B shows the measured GSH levels. Compared with the control group, the 40 μM IMP group showed a 1.446-fold increase in GSH level. We observed that supplementation of 40 μM IMP to IVM medium significantly improved the redox balance of mouse oocytes (Figure 1C; 1 ± 0.34 vs. 1.44 ± 0.11-fold).

### 3.2. Effects of IMP Supplementation on IVF of Vitrified Mouse Oocytes

We investigated whether the addition of different concentrations of IMP during the vitrification process improved the developmental capacity of post-thawed mouse oocytes. Our results demonstrated that the fertilization rate of post-thawed oocytes in the 40 μM IMP group during IVF was significantly higher than that of the vitrification (0 μM IMP) (Table 3; 44.17% (±1.56%) vs. 31.75% (±1.5%)). In addition, we found that the addition of 40 μM IMP to the vitrified freezing solution significantly enhanced the survival of thawed oocytes (Table 4; 66.41% (±2.07%) vs. 76.91% (±2.12%)). Based on these findings, we chose a concentration of 40 μM IMP for further experiments.

### 3.3. Effect of IMP Supplementation During Vitrification–Thawing on the Hydrolysis Resistance of Mouse Oocyte Zona Pellucida and the Distribution of Cortical Granules

**Figure 2:**
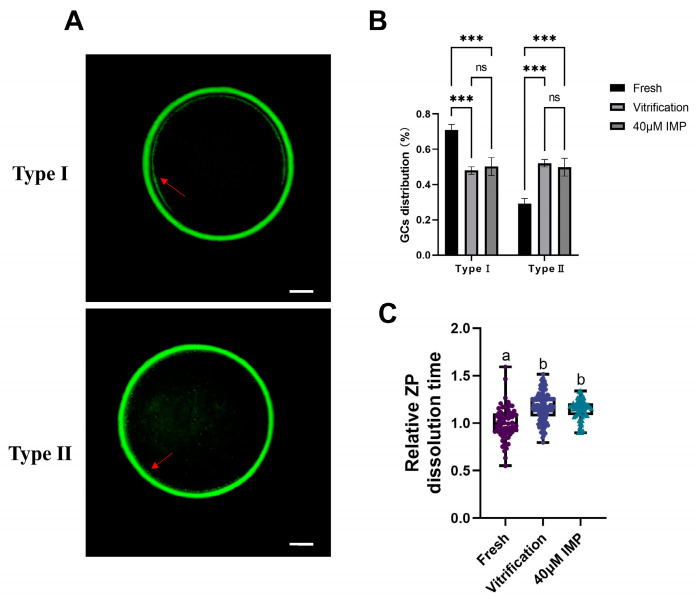
Effects of imperatorin (IMP) supplementation on cortical granule distribution and zona pellucida (ZP) hardening in vitrified–thawed mouse oocytes. (**A**) Representative cortical granule distributions; arrows indicate cortical particles, scale = 25 μm for Type I and Type II; (**B**) cortical granule distributions of oocytes of the fresh group (*n* = 76), vitrification group (*n* = 69), and 40 μM IMP group (*n* = 82), ***: *p* < 0.001; (**C**) effects of IMP supplementation during vitrification on the degree of ZP hardening in metaphase II (MII) oocytes of fresh group (*n* = 72), vitrification group (*n* = 139), and 40 μM IMP group (*n* = 62). Different lowercase letters denote significant differences (*p* < 0.05).

Vitrification–thawing exerts an influence on the morphological structures of the oocytes, thereby reducing ovum IVF efficiency. Therefore, we performed staining of cortical granules to observe the effects of IMP supplementation on vitrification–thawing. Type I: almost all cortical granules are distributed in the cortex and form a continuous halo around the plasma membrane; Type II: cortical granules are more distributed in the cytoplasm than at the plasma membrane. Premature cortical granule release leads to ZP hardening, which reduces the efficiency of IVF. Our results indicated that vitrified–thawed mouse oocytes exhibited a greater degree of disorderliness in cortical granule distribution compared with the fresh group (Figure 2B; 70.96% (±2.69%) vs. 47.94% (±2.11%)). The addition of IMP to the vitrification medium did not improve the distribution of cortical granules.

Given that ZP hardening enhances resistance against proteolytic digestion [35,36,37], we examined the degree of ZP hardening by digesting ZP with pronase. Compared with the positive control group, the addition of IMP to the vitrification medium did not alter the degree of ZP hardening. A video of the hydrolysis of the transparent tape can be found in Video S1.

### 3.4. Effects of IMP Supplementation During Vitrification–Thawing on Oxidative Stress in Mouse Oocytes

**Figure 3:**
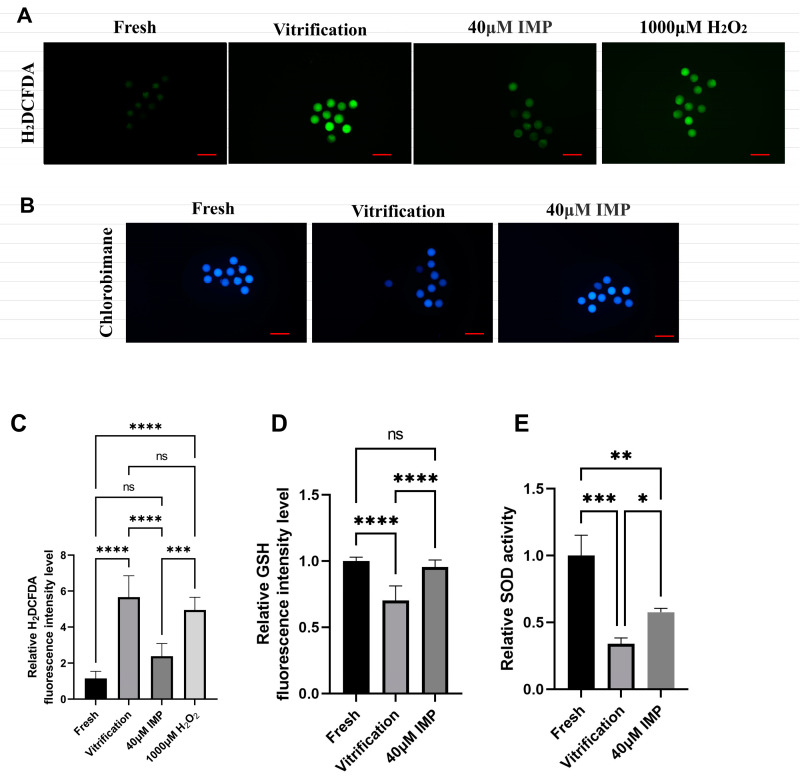
Effects of imperatorin (IMP) supplementation during vitrification on oxidative stress levels in MII mouse oocytes. (**A**) Representative fluorescence images of intracellular reactive oxygen species (ROS) staining in mouse oocytes of the control and IMP supplementation groups, scale = 100 μm; (**B**) representative images of glutathione (GSH) staining in mouse oocytes of the different groups; (**C**) quantified relative ROS level of the fresh group (*n* = 60), vitrification group (*n* = 90), and 40 μM IMP group (*n* = 70), R = 6; (**D**) quantified relative GSH level of the fresh group (*n* = 80), vitrification group (*n* = 70), and 40 μM IMP group (*n* = 80), R = 7; (**E**) relative superoxide dismutase (SOD) activity of the different treatment groups (*n* = 450), R = 3, scale = 100 μm. *: *p* < 0.05; **: *p* < 0.01; ***: *p* < 0.001; ****: *p* < 0.0001.

**Figure 4:**
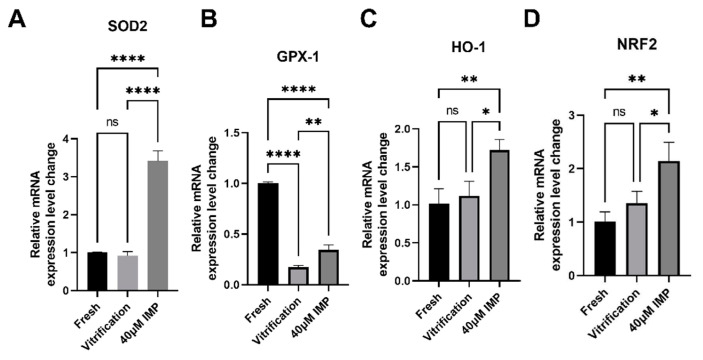
Effects of imperatorin (IMP) on antioxidant gene transcription in vitrified–thawed oocytes. (**A**–**D**) Differential gene expression in oocytes of the fresh group, vitrification group, and 40 μM IMP group. Gene expression in the oocytes was measured at 2 h after vitrification–thawing. R = 3. *: *p* < 0.05; **: *p* < 0.01. ****: *p* < 0.0001.

Oxidative stress is one of the most severe problems faced by vitrified–thawed mouse oocytes. To investigate whether IMP reduced the oxidative stress level after vitrification–thawing, we used H_2_DCFDA as a fluorescent probe for measuring the ROS levels of cells and established a model of oxidatively stressed oocytes using different concentrations of H_2_O_2_ (Figure S1). The relative fluorescence level of the 40 μM IMP group was significantly lowered compared with the positive control group (Figure 3C; 2.39 ± 0.89 vs. 5.66 ± 1.20) but still higher than that of the negative control group (2.39 ± 0.89 vs. 1 ± 0.29). The SOD activity of the 40 μM IMP group was significantly higher than that of the vitrification group (Figure 3E; 0.58 ± 0.05 vs. 0.34 ± 0.04). This is consistent with the qPCR results (Figure 4)indicating that IMP treatment leads to inhibition of SOD2 and GPX1 mRNA degradation in thawed mouse oocytes. Measurement of GSH levels by the mBBr probe revealed that the addition of IMP to vitrification medium enhanced the GSH content within oocytes (95.59 ± 7.34% vs. 70.21 ± 14.36%). The GSH level of the 40 μM IMP group was still lower than that of the fresh group, although the difference was not statistically significant.

### 3.5. IMP Supplementation Inhibited Apoptosis of Vitrified–Thawed Mouse Oocytes

**Figure 5:**
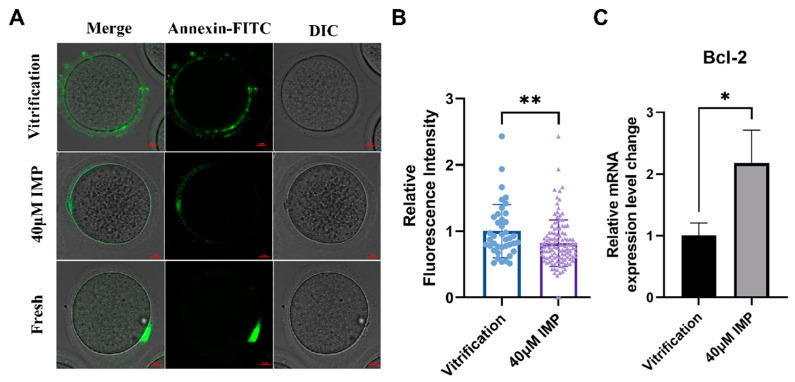
Effects of imperatorin (IMP) supplementation during vitrification on apoptotic levels in MII mouse oocytes. (**A**) Representative images of Annexin-V staining, scale = 25 μm; (**B**) area of positive Annexin-V-FITC staining in mouse oocytes of the vitrification group (*n* = 40) and 40 μM IMP group (*n* = 76); (**C**) relative Bcl-2 mRNA level of the fresh group, vitrification group, and 40 μM IMP group, scale = 25 μm. *: *p* < 0.05; **: *p* < 0.01.

To assess whether IMP regulated apoptotic levels in vitrified–thawed oocytes, we measured the externalization of phosphatidylserine in the oocytes by Annexin-V-FITC staining and determined the fluorescence intensities of the various groups. Our results revealed that the area of positive fluorescence in the 40 μM IMP group was significantly smaller than that of the vitrification group (Figure 5; 2.26 ± 1.49 vs. 4.67 ± 2.29). Compared with the vitrification group with 40 μM IMP, the Bcl-2 transcript level in the vitrification group was degraded 2.17 (±0.577) fold, which was consistent with the qPCR results.

### 3.6. Effects of Addition of IMP to Vitrification and Thawing Media on Mitochondria

**Figure 6:**
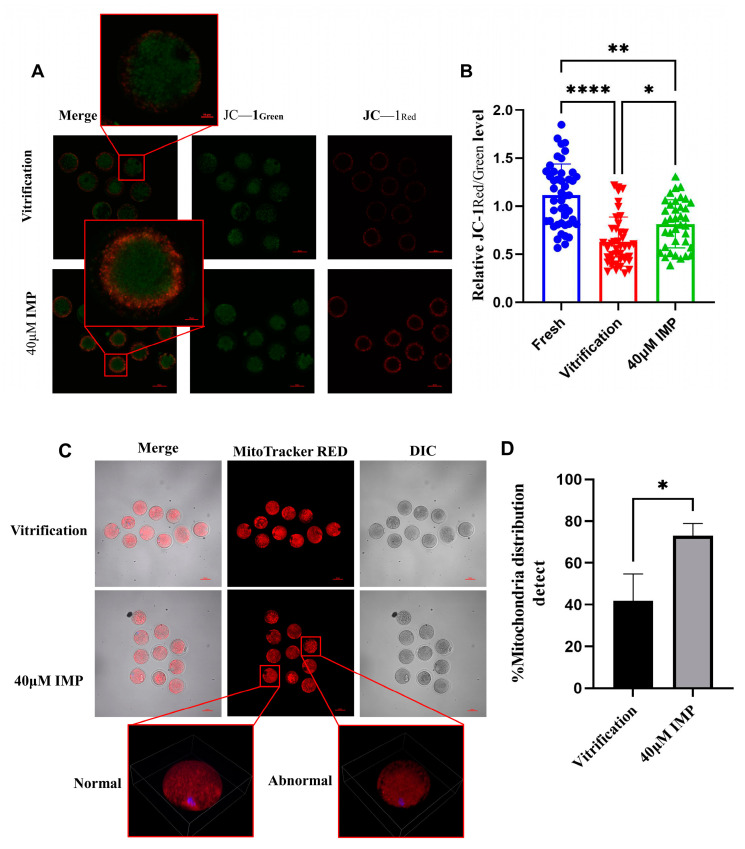
Effects of imperatorin (IMP) supplementation during vitrification–thawing on mitochondria of metaphase II (MII) oocytes. (**A**) Representative fluorescence images of JC-1 staining of mitochondria in mouse oocytes, scale = 50 μm; (**B**) ratio of JC-1 aggregate fluorescence (red)/monomer fluorescence (green) of the fresh group (*n* = 45), vitrification group (*n* = 42), and 40 μM IMP group (*n* = 37); (**C**) representative MitoTracker^TM^ Deep Red fluorescence image of two-dimensional mitochondrial distribution in oocytes, scale = 50 μm; three-dimensional mitochondrial distribution in oocytes, where “Normal” and “Abnormal” indicates the representative image for post-vitrified oocytes; (**D**) mitochondrial distribution levels in mouse oocytes of the vitrification group (*n* = 40) and 40 μM IMP group (*n* = 40).; *: *p* < 0.05; **: *p* < 0.01. ****: *p* < 0.0001.

Mitochondria are crucial for the maintenance of oocyte quality. The effects of IMP on the mitochondria of vitrified–thawed mouse oocytes were assessed through an investigation of MMP and mitochondrial distribution. Staining was performed using JC-1, which originally exists as monomers emitting green fluorescence and forms aggregates that emit red fluorescence in mitochondria under the influence of MMP. Mitochondrial health was subsequently determined based on the ratio of red fluorescence intensity to green fluorescence intensity. Our results show that the average MMP levels of the vitrification group and 40 μM IMP group compared with the fresh group were 63% and 79.61%, respectively. Therefore, the addition of 40 μM IMP to vitrification media significantly improved MMP compared with vitrification without IMP (Figure 6B; 79.61% vs. 63.04%).

Although the distribution of mitochondria in oocytes of different periods is characterized by separate features, for MII-stage oocytes, the dispersed cytoplasmic distribution of mitochondria is a hallmark of normal cellular organization [38]. The distribution patterns of mitochondria are associated with their energy supply efficiency and play a key role in the development of fertilized embryos [39]. In the present study, mitochondrial distribution was detected using MitoTracker^TM^ Deep Red (Thermo Fisher Scientific, Waltham, MA, USA). From the three-dimensional and two-dimensional distributions of the mitochondria (Figure 6C), it can be observed that the addition of IMP to the vitrification medium improved mitochondrial distribution to a certain extent compared with the vitrification group (72.95% ± 2.95% vs. 41.875% ± 18.125%).

**Figure 7:**
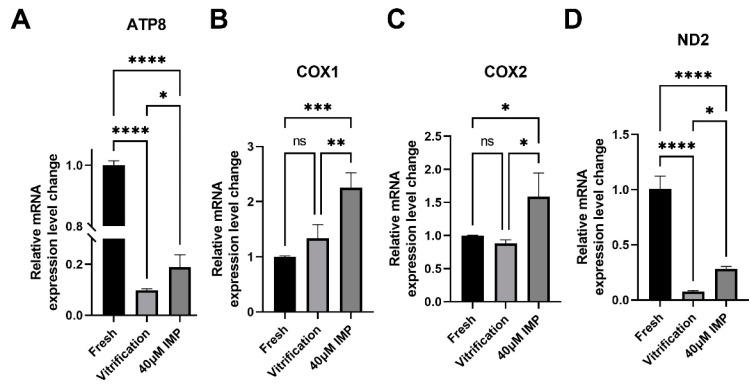
Effects of imperatorin (IMP) supplementation during vitrification–thawing on mitochondrial mRNA levels of MII oocytes. (**A**–**D**) Relative mRNA levels of ND2, ATP8, COX2, COX1, NRF2, and HO-1 of the fresh group, vitrification group, and 40 μM IMP group. *: *p* < 0.05; **: *p* < 0.01.***: *p* < 0.001.****: *p* < 0.0001.

In light of the lower MMP observed in the vitrified–thawed mouse oocytes, we analyzed the expression of mitochondrial respiratory chain genes in post-thawed oocytes that had been vitrified with the addition of IMP in the vitrification medium. Results of qPCR revealed that, compared with oocytes without IMP addition, the IMP-modified vitrification medium significantly affected the transcription levels of ND2, ATP8, COX2, and COX1 (Figure 7). This was consistent with the results of MMP measurement, further demonstrating that the addition of IMP to the vitrification medium improved MMP through upregulation of the relevant genes.

## 4. Discussion

In vitro maturation (IVM) of the oocytes, which involves maturation of both the nucleus and cytoplasm, is one of the key steps of in vitro embryonic production. Compared with maturation in vivo, IVM is subject to the effects of external factors such as light exposure, high environmental oxygen concentration, pH, and temperature changes, which leads to reduced maturation efficiency. Therefore, we investigated the effects of supplementing the IVM culture medium with imperatorin (IMP), a main bioactive component of certain traditional Chinese herbs. Our results indicate that IMP promoted first polar body (PB1) extrusion. This is in agreement with the findings of previous research, which demonstrated that the addition of an antioxidant during the IVM process enhanced ovum maturation efficiency [40,41,42]. Moreover, we found that IMP reduced the ROS level and increased the GSH level in oocytes, which may be the main reason for the promotion of ovum maturation and improvement of ovum quality. Given the antioxidant properties of IMP and the critical issue of post-thaw oxidative stress faced by vitrified–thawed mouse oocytes, we attempted to investigate the effects of adding IMP to the vitrification and thawing media.

Vitrification freezing technique is an efficient method of oocyte preservation, but its negative impact on oocyte fertilization rate and subsequent developmental capacity during freezing and rewarming has become an important issue. Our experimental results presented a decrease in fertilization ability as well as developmental capacity of oocytes after vitrification freezing. In agreement with previous studies [43], the in vitro fertilization ability of oocytes after vitrification freezing was significantly reduced. For this reason, we investigated how IMP can improve oocyte status to enhance fertilization.

Currently, vitrification technology for ovum cryopreservation offers a plethora of advantages compared with slow freezing. Nakagata et al. [44] reported that vitrified mouse oocytes achieved a relatively high survival rate after thawing. In the present study, the vitrified–thawed mouse oocytes exhibited a survival rate of 66.41% after 3 h of placement in M16 medium. This indicates that vitrification technology provides greater reliability, but issues related to the subsequent development of the post-thawed oocytes will remain a key focus area in pertinent research.

It is widely known that ROS will exert a considerable influence on cellular structure when its generation exceeds the maximum ROS elimination capacity of the antioxidant defense system. In vivo, oocytes can defend against excessive ROS through free radical scavengers in the cytoplasm and surrounding environment, such as the ovarian follicles and fallopian tubal fluid. However, oocytes in vitro, especially vitrified oocytes, are particularly sensitive to ROS due to reduced antioxidant activity within thawed cells and the lack of free radical scavengers in the extracellular environment [45]. Our results also indicated that the SOD activity of vitrified–thawed oocytes was reduced by 66%, while the. ROS level was increased by 5.6 ± 2.3-fold compared with fresh oocytes. We also found that vitrification lowered the level of the nonenzymatic antioxidant GSH. Additionally, qPCR revealed that IMP rescued the mRNA level of *GPX1*. It is known that GPX1 decomposes H_2_O_2_ by oxidizing two GSHs to produce GSSG [46]. Similarly, the low mRNA levels of *SOD2* in the vitrification freezing group compared to the IMP group may be due to the degradation of mRNA as a result of increased ROS levels [47]. So much so that it eventually showed low SOD activity compared to the IMP group.

The reduction in ROS is a key step in enhancing vitrification efficiency. A study by Liao et al. [48] reported that IMP reduced the oxidative stress level of hippocampal neurons in a cobalt(II)-chloride-induced model of vascular dementia. IMP also exhibited strong antioxidant properties in a mouse model of Parkinson’s disease, which were manifested as the ability to regulate the PI3K/Akt pathway and improve cognitive function in mice. Another study showed that IMP reduced in vitro aging-induced oxidative stress in porcine oocytes [49]. Consistent with the studies described above, the present study has demonstrated that IMP possesses strong antioxidant properties, which are manifested through *NRF2*. Transcriptional activation of *HO-1* responds to various chemical and physical stimuli such as oxidants and phytochemical antioxidants [50], as demonstrated by our qPCR results. We found that the addition of 40 μM IMP to the vitrification medium significantly reduced the ROS level, increased the GSH level, and attenuated the influence of vitrification on antioxidant enzyme activities within mouse oocytes. In summary, also considering the IVF-related results, it can be deduced that vitrification-induced oxidative stress was at least partially responsible for poor embryonic development following fertilization of vitrified oocytes.

Moreover, vitrification can cause premature exocytosis of cortical granules, which leads to the hardening of ZP [51]. Previous research has contended that disulfide bonds enable the maintenance of ZP structure and are the cause of ZP hardening [52]. Our results (Figure 2) revealed that vitrification led to premature ZP hardening, which also explains the significant decrease of 39.63% in the fertilization rate of vitrified–thawed oocytes compared with fresh oocytes. This issue can be resolved by intracytoplasmic sperm injection (ICSI) in the oocytes.

In the present study, we found that the addition of IMP to vitrification medium improved MMP and mitochondrial distribution in oocytes while reducing the ROS level, which explained the significant increase in fertilization rate to a certain extent. ATP depletion and cytoskeletal damage caused by mitochondrial dysfunction and ROS are among the main factors driving reduced fertilization in oocytes [53]. Mitochondria are usually uniformly distributed in the cytoplasm of mature oocytes [54]. Our results showed that the addition of IMP to vitrification medium improved mitochondrial distribution in mouse oocytes. Given that MitoTracker staining is dependent on MMP, it is likely that IMP influences mitochondrial distribution through the improvement of MMP. However, further research will be necessary for confirmation of this conjecture. We also observed that vitrification led to a decrease in MMP. This is a manifestation of early apoptosis and corroborates the findings of phosphatidylserine staining, i.e., vitrification induced apoptosis in the oocytes. However, the addition of 40 μM IMP significantly inhibited the early apoptosis of oocytes (Figure 5). IMP is a small-molecule compound that can penetrate the cell membrane and restore the MMP of post-vitrified cells. COX2, COX1, and ATP8 are components of the oxidative respiratory chain, with ATP8 responsible for the generation of ATP [55].

Although IMP was able to reduce ROS levels and enhance in vitro fertilization, it still failed to improve blastocyst rates and vitrification can cause various types of damage to oocytes, such as ultrastructural changes [56], low-temperature-induced spindle damage [57,58], impairment of DNA integrity [59], and epigenetic perturbations [60,61]. We believe that other forms of damage caused by vitrification cause the inability for safe development of oocytes to the blastocyst stage. Therefore, modifications to the vitrification technology may be a key approach to the enhancement of vitrification efficiency [62,63,64,65]. This is of great significance for the preservation of excellent species genetic resources.

## 5. Conclusions

In summary, IMP supplementation improved the quality of vitrification-frozen mouse oocytes and their subsequent fertilizing ability by increasing antioxidant capacity, decreasing ROS accumulation, failing to rescue premature release of cortical particles, preventing abnormalities in mitochondrial distribution, enhancing mitochondrial function, and inhibiting the onset of apoptosis; however, the developmental ability of vitrification-frozen oocytes for improved post-fertilization development still did not play a role. These findings provide a theoretical basis for improving animal reproduction and in vitro embryo production.

## Figures and Tables

**Table 1:** Primer design.

Name	Gene ID	Sequences r	Tm
GAPDH	14433	F-TCTTGCTCAGTGTCCTTGC	58.00
		R-CTTTGTCAAGCTCATTTCCTGG	56.26
Mt-ND2	5912278	F-TTCGTCACACAAGCAACAGC	55.40
		R-GGGGCGAGGCCTAGTTTTAT	57.45
Mt-COX2	5912281	F-CCTCCACTCATGAGCAGTCC	57.45
		R-AACCCTGGTCGGTTTGATGTT	55.85
Mt-ATP8	5912284	F-CACAAACATTCCCACTGGCAC	57.80
		R-TTGGGGTAATGAATGAGGCAAA	54.40
Mt-COX1	5912286	F-TCGGAGCCCCAGATATAGCA	57.45
		R-TTTCCGGCTAGAGGTGGGTA	57.45
NRF2	18024	F-GACTACAGTCCCAGCAGAGTG	59.76
		R-TCTGCGTGCTCAGAAACCTC	59.45
BCL-2	12043	F-GACTGAGTACCTGAACCGGC	59.50
		R-AGTTCCACAAAGGCATCCCAG	57.80
GPX-1	14775	F-AGTCCACCGTGTATGCCTTCT	57.80
		R-GAGACGCGACATTCTCAATGA	55.85
HO-1	15368	F-TGAATCGAGCAGAACCAGCC	57.45
		R-CAAATCCTGGGGCATGCTGTC	59.76

**Table 2:** Oocytes imperatorin (IMP) in mice at GV stage.

	Total Number	PBE
Control	108 (±12)	55.13% (±5.13%) ^b^
20 μM IMP	89 (±1)	51.36% (±6.31%) ^b^
40 μM IMP	108 (±11)	69.79% (±1.75%) ^a^
60 μM IMP	102 (±17)	50.73% (±1.71%) ^b^

Different letters meant there was significant difference among groups (*p* < 0.05).

**Table 3:** In vitro fertilization (IVM) of vitrified mouse oocytes.

	Total Number	Fertilization Rate	Blastocyst Rate
Fresh	132 (±20)	75.38% (±4.48%) ^a^	54.22% (±8.07%) ^a^
Vitrification—COCs	105 (±11.3)	36.80% (±6.56%) ^bc^	9.29% (±2.90%) ^b^
Vitrification (0 μM IMP)	119 (±0.33)	31.75% (±1.5%) ^c^	3.35% (±1.98%) ^b^
20 μM IMP	101 (±7)	31.18% (±0.91%) ^c^	5.79% (±0.89%) ^b^
40 μM IMP	114 (±14)	44.17% (±1.56%) ^b^	6.75% (±1.33%) ^b^
60 μM IMP	107 (±4)	21.54% (±3.94%) ^d^	2.07 (±1.57%) ^b^

Negative control: fresh group. Positive control: vitrification (0 μM IMP). Vitrification—COCs grouped as oocytes with cumulus cells. IMP: imperatorin. Different letters meant there was significant difference among groups (*p* < 0.05).

**Table 4:** Viability of vitrified–thawed mouse oocytes.

	Total Number	Survival Rate
Vitrification	110 (±8)	66.41% (±2.07%) ^a^
40 μM imperatorin	112 (±7)	76.91% (±2.12%) ^b^

Different letters meant there was significant difference among groups (*p* < 0.05).

## Data Availability

The original contributions presented in this study are included in the article/Supplementary Material. Further inquiries can be directed to the corresponding author.

## Supplementary Materials

The following supporting information can be downloaded at: https://www.mdpi.com/article/10.3390/ani15050661/s1, Figure S1: Modeling of oxidative stress in oocytes with different concentrations of hydrogen peroxide; Video S1: Enzymatic digestion of zona pellucida.

## References

[1] Chang C.-C., Shapiro D.B., Nagy Z.P. (2022). The effects of vitrification on oocyte quality. Biol. Reprod..

[2] Mandawala A.A., Harvey S.C., Roy T.K., Fowler K.E. (2016). Cryopreservation of animal oocytes and embryos: Current progress and future prospects. Theriogenology.

[3] Kopeika J., Thornhill A., Khalaf Y. (2015). The effect of cryopreservation on the genome of gametes and embryos: Principles of cryobiology and critical appraisal of the evidence. Hum. Reprod. Update.

[4] Park S.E., Hwang W.S., Lim J.M. (2001). Cryopreservation of ICR mouse oocytes: Improved post-thawed preimplantation development after vitrification using Taxol^TM^, a cytoskeleton stabilizer. Fertil. Steril..

[5] Akin J.W., Bell K.A., Thomas D., Boldt J. (2007). Initial experience with a donor egg bank. Fertil. Steril..

[6] Bernard A. (1996). Cryopreservation of human oocytes: A review of current problems and perspectives. Hum. Reprod. Update.

[7] Len J.S., Koh WS D., Tan S.-X. (2019). The roles of reactive oxygen species and antioxidants in cryopreservation. Biosci. Rep..

[8] Zorov D.B., Filburn C.R., Klotz L.-O., Zweier J.Z., Sollott S.J. (2000). Reactive Oxygen Species (ROS)-induced ROS Release: A New Phenomenon Accompanying Induction of the Mitochondrial Permeability Transition in Cardiac Myocytes. J. Exp. Med..

[9] Xu X., Cowley S., Flaim C.J., James J., Seymour L., Cui Z.F. (2010). The roles of apoptotic pathways in the low recovery rate after cryopreservation of dissociated human embryonic stem cells. Biotechnol. Prog..

[10] Hockberger P.E., Skimina T.A., Centonze V.E., Lavin C., Chu S., Dadras S., Reddy J.K., White J.G. (1999). Activation of flavin-containing oxidases underlies light-induced production of H_2_O_2_ in mammalian cells. Proc. Natl. Acad. Sci. USA.

[11] Squirrell J.M., Wokosin D.L., White J.G., Bavister B.D. (1999). Long-term two-photon fluorescence imaging of mammalian embryos without compromising viability. Nat. Biotechnol..

[12] Ottosen LD M., Hindkjær J., Ingerslev J. (2007). Light exposure of the ovum and preimplantation embryo during ART procedures. J. Assist. Reprod. Genet..

[13] Guerin P. (2001). Oxidative stress and protection against reactive oxygen species in the pre-implantation embryo and its surroundings. Hum. Reprod. Update.

[14] Birben E., Sahiner U.M., Sackesen C., Erzurum S., Kalayci O. (2012). Oxidative Stress and Antioxidant Defense. World Allergy Organ. J..

[15] Chang H., Chen H., Zhang L., Wang Y., Xie X., Zhang Y., Quan F.S. (2019). Effect of oocyte vitrification on DNA damage in metaphase II oocytes and the resulting preimplantation embryos. Mol. Reprod. Dev..

[16] Zribi N., Feki Chakroun N., El Euch H., Gargouri J., Bahloul A., Keskes L.A. (2010). Effects of cryopreservation on human sperm deoxyribonucleic acid integrity. Fertil. Steril..

[17] Borjizadeh A., Ahmadi H., Daneshi E., Roshani D., Fathi F., Abdi M., Nasseri S., Abouzaripour M. (2019). The effect of adding Rosmarinic and Ascorbic acids to vitrification media on fertilization rate of the mice oocyte: An experimental study. Int. J. Reprod. BioMed..

[18] Wang Y., Zhang M., Chen Z.-J., Du Y. (2018). Resveratrol promotes the embryonic development of vitrified mouse oocytes after in vitro fertilization. Vitr. Cell. Dev. Biol. Anim..

[19] Li Z., Gu R., Lu X., Zhao S., Feng Y., Sun Y. (2018). Preincubation with glutathione ethyl ester improves the developmental competence of vitrified mouse oocytes. J. Assist. Reprod. Genet..

[20] Zhang Y.-B., Deng G.-G., Wang T.-X., Liu L., Yang X.-W. (2019). Tissue distribution study of *Angelica dahurica* cv. Yubaizhi in rat by ultra–performance liquid chromatography with tandem mass spectrometry. J. Pharm. Biomed. Anal..

[21] Kwon M.H., Jeong J.S., Ryu J., Cho Y.W., Kang H.E. (2017). Simultaneous determination of saikosaponin a, paeonol, and imperatorin, components of DA-9805, in rat plasma by LC–MS/MS and application to a pharmacokinetic study. J. Chromatogr. B.

[22] Kerekes D., Csorba A., Gosztola B., Zámbori E.N., Kiss T., Csupor F. (2019). Furocoumarin Content of Fennel—Below the Safety Threshold. Molecules.

[23] Chen X.-P., Li W., Xiao X.-F., Zhang L.-L., Liu C.-X. (2013). Phytochemical and pharmacological studies on Radix Angelica sinensis. Chin. J. Nat. Med..

[24] Sigurdsson S., Gudbjarnason S. (2013). Effect of oral imperatorin on memory in mice. Biochem. Biophys. Res. Commun..

[25] Kozioł E., Skalicka-Woźniak K. (2016). Imperatorin–pharmacological meaning and analytical clues: Profound investigation. Phytochem. Rev..

[26] Cao Y.-J., He X., Wang N., He L.-C. (2013). Effects of imperatorin, the active component from Radix Angelicae (Baizhi), on the blood pressure and oxidative stress in 2K,1C hypertensive rats. Phytomedicine.

[27] Budzynska B., Boguszewska-Czubara A., Kruk-Slomka M., Michalak A., Musik I., Biala G. (2015). Effects of imperatorin on scopolamine-induced cognitive impairment and oxidative stress in mice. Psychopharmacology.

[28] Ahmad N., Ansari M., Haqqi T. (2019). Imperatorin, a plant derived small molecule, inhibits oxidative stress and prevents mitochondrial damage in human oa chondrocytes. Osteoarthr. Cartil..

[29] Takeo T., Nakagata N. (2011). Reduced Glutathione Enhances Fertility of Frozen/Thawed C57BL/6 Mouse Sperm after Exposure to Methyl-Beta-Cyclodextrin1. Biol. Reprod..

[30] Takeo T., Nakagata N. (2010). Combination medium of cryoprotective agents containing l -glutamine and methyl-*β*-cyclodextrin in a preincubation medium yields a high fertilization rate for cryopreserved C57BL/6J mouse sperm. Lab. Anim..

[31] Chao S., Li L.-J., Lu J., Zhao S.-X., Zhao M.-H., Huang G.-A., Yin S., Shen W., Sun Q.-Y., Zhao Y. (2023). Epigallocatechin gallate improves the quality of diabetic oocytes. Biomed. Pharmacother..

[32] Abedpour N., Rajaei F. (2015). Vitrification by Cryotop and the Maturation, Fertilization, and Developmental Rates of Mouse Oocytes. Iran. Red Crescent Med. J..

[33] Coy P., Grullon L., Canovas S., Romar R., Matas C., Aviles M. (2008). Hardening of the zona pellucida of unfertilized eggs can reduce polyspermic fertilization in the pig and cow. Reproduction.

[34] Nikishin D.A. (2018). Selection of stable expressed reference genes in native and vitrified/thawed human ovarian tissue for analysis by qrt-pcr and western blot. J. Assist. Reprod. Genet..

[35] Barros C., Yanagimachi R. (1971). Induction of Zona Reaction in Golden Hamster Eggs by Cortical Granule Material. Nature.

[36] Austin C.R., Braden A.W.H. (1956). Early Reactions of the Rodent Egg to Spermatozoon Penetration. J. Exp. Biol..

[37] Gulyas B.J., Yuan L.C. (1985). Cortical reaction and zona hardening in mouse oocytes following exposure to ethanol. J. Exp. Zool..

[38] Hao X., Zhao J., Rodriguez-Wallberg K.A. (2024). Comprehensive atlas of mitochondrial distribution and dynamics during oocyte maturation in mouse models. Biomark. Res..

[39] Kirillova A., Smitz J.E.J., Sukhikh G.T., Mazunin I. (2021). The Role of Mitochondria in Oocyte Maturation. Cells.

[40] Banihosseini S.Z., Novin M.G., Nazarian H., Piryaei A., Parvardeh S., Eini F. (2018). Quercetin improves developmental competence of mouse oocytes by reducing oxidative stress during in vitro maturation. Ann. Anim. Sci..

[41] Rostami T., Fathi F., Assadollahi V., Hosseini J., Erfan M.B.K., Rashidi A., Amiri G., Banafshi O., Alasvand M. (2021). Effect of cyanocobalamin on oocyte maturation, in vitro fertilization, and embryo development in mice. Zygote.

[42] Amatyakul P., Kruevaisayawan H., Khongsombat O., Yamtanodea S. (2017). Effects of lycopene on mouse sperm and oocyte, and in vitro fertilization outcomes. ScienceAsia.

[43] Abedpour N., Shoorei H., Rajaei F. (2023). Detrimental effects of vitrification on integrin genes (α9 and β1) and in vitro fertilization in mouse oocytes. Mol. Biol. Rep..

[44] Nakagata N. (1989). High survival rate of unfertilized mouse oocytes after vitrification. Reproduction.

[45] Habibi A., Farrokhi N., Moreira Da Silva F., Bettencourt B.F., Bruges-Armas J., Amidi F., Hosseini A. (2010). The effects of vitrification on gene expression in mature mouse oocytes by nested quantitative PCR. J. Assist. Reprod. Genet..

[46] Lubos E., Loscalzo J., Handy D.E. (2011). Glutathione Peroxidase-1 in Health and Disease: From Molecular Mechanisms to Therapeutic Opportunities. Antioxid. Redox Signal..

[47] Yang W.-H., Bloch D.B. (2007). Probing the mRNA processing body using protein macroarrays and “autoantigenomics”. RNA.

[48] Liao X., Zhang Z., Ming M., Zhong S., Chen J., Huang Y. (2023). Imperatorin exerts antioxidant effects in vascular dementia via the Nrf2 signaling pathway. Sci. Rep..

[49] Luo D., Zhang J., Li S., Liu W., Yao X.-R., Guo H., Jin Z.-L., Jin Y.-X., Yuan B., Jiang H. (2020). Imperatorin Ameliorates the Aging-Associated Porcine Oocyte Meiotic Spindle Defects by Reducing Oxidative Stress and Protecting Mitochondrial Function. Front. Cell Dev. Biol..

[50] Ryter S.W. (2022). Heme Oxygenase-1: An Anti-Inflammatory Effector in Cardiovascular, Lung, and Related Metabolic Disorders. Antioxidants.

[51] Larman M.G., Sheehan C.B., Gardner D.K. (2006). Calcium-free vitrification reduces cryoprotectant-induced zona pellucida hardening and increases fertilization rates in mouse oocytes. Reproduction.

[52] Kwamoto K., Ikeda K., Yonezawa N., Noguchi S., Kudo K., Hamano S., Kuwayama M., Nakano M. (1999). Disulfide formation in bovine zona pellucida glycoproteins during fertilization: Evidence for the involvement of cystine cross-linkages in hardening of the zona pellucida. Reproduction.

[53] Ou X.-H., Li S., Wang Z.-B., Li M., Quan S., Xing F., Guo L., Chao S.-B., Chen Z., Liang X.-W. (2012). Maternal insulin resistance causes oxidative stress and mitochondrial dysfunction in mouse oocytes. Hum. Reprod..

[54] Kandil O.M., Rahman SM A.E., Ali R.S., Ismail E.A., Ibrahim N.M. (2024). Effect of melatonin on developmental competence, mitochondrial distribution, and intensity of fresh and vitrified/thawed in vitro matured buffalo oocytes. Reprod. Biol. Endocrinol..

[55] Barshad G., Marom S., Cohen T., Mishmar D. (2018). Mitochondrial DNA Transcription and Its Regulation: An Evolutionary Perspective. Trends Genet..

[56] Khalili M.A., Shahedi A., Ashourzadeh S., Nottola S.A., Macchiarelli G., Grazia M. (2017). Vitrification of human immature oocytes before and after in vitro maturation: A review. J. Assist. Reprod. Genet..

[57] Van Der Elst J., Van Den Abbeel E., Jacobs R., Wisse E., Steirteghem A.V. (1988). Effect of 1,2-propanediol and dimethylsulphoxide on the meiotic spindle of the mouse oocyte. Hum. Reprod..

[58] Keefe D., Liu L., Wang W., Silva C. (2003). Imaging meiotic spindles by polarization light microscopy: Principles and applications to IVF. Reprod. BioMed. Online.

[59] Berthelot-Ricou A., Perrin J., Di Giorgio C., Meo D., Botta A., Courbiere B. (2013). Genotoxicity assessment of mouse oocytes by comet assay before vitrification and after warming with three vitrification protocols. Fertil. Steril..

[60] Yan L.-Y., Yan J., Qiao J., Zhao P.-L., Liu P. (2010). Effects of oocyte vitrification on histone modifications. Reprod. Fertil. Dev..

[61] Cheng K.-R., Fu X.-W., Zhang R.-N., Jia J.-X., Hou Y.-P., Zhu S.-E. (2014). Effect of oocyte vitrification on deoxyribonucleic acid methylation of H19, Peg3, and Snrpn differentially methylated regions in mouse blastocysts. Fertil. Steril..

[62] Abdel-Halim B.R., Helmy N.A. (2018). Effect of nano-selenium and nano-zinc particles during in vitro maturation on the developmental competence of bovine oocytes. Anim. Prod. Sci..

[63] Cao X.-Y., Rose J., Wang S.-Y., Liu Y., Zhao M., Xing M.-J., Chang T., Xu B. (2016). Glycine increases preimplantation development of mouse oocytes following vitrification at the germinal vesicle stage. Sci. Rep..

[64] Davoodian N., Kadivar A., Ahmadi E., Nazari H., Mehrban H. (2021). Quercetin effect on the efficiency of ovine oocyte vitrification at GV stage. Theriogenology.

[65] Sprícigo J.F., Morató R., Arcarons N., Yeste M., Dode M.A., López-Bejar M., Mogas T. (2017). Assessment of the effect of adding L-carnitine and/or resveratrol to maturation medium before vitrification on in vitro-matured calf oocytes. Theriogenology.

