# Supplementary figures and images for: Effects and Mechanisms of Imperatorin on Vitrified Mouse Oocytes
Source: Animals (Basel). 2025 Feb 25;15(5):661. doi: 10.3390/ani15050661 (PMC11898152; doi:10.3390/ani15050661)

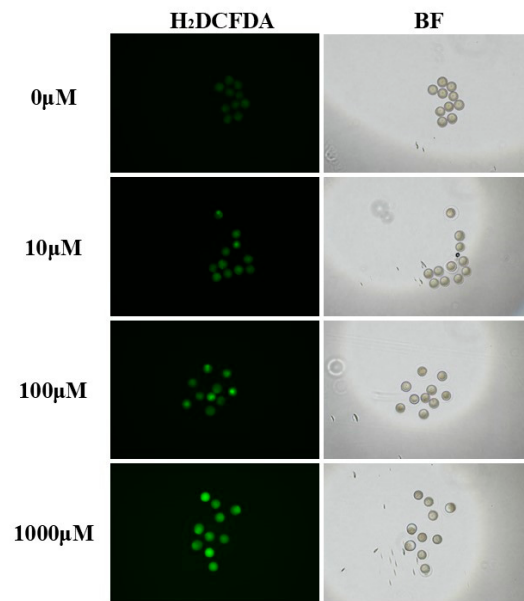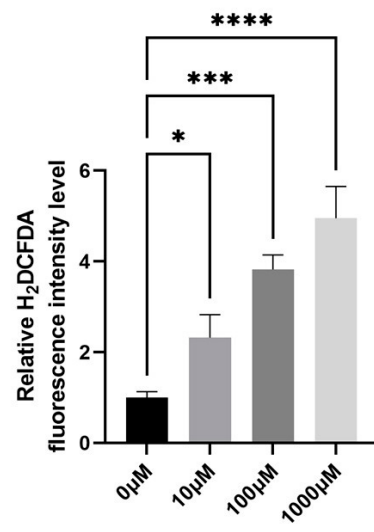

Supplement: Supplementary file 1 [file animals-15-00661-s001.zip › S1 Modeling of oxidative stress in oocytes with different concentrations of hydrogen peroxide.pdf]
